# Defying the Odds: Survival in Severe Prenatal Caffey's Disease

**DOI:** 10.7759/cureus.98173

**Published:** 2025-11-30

**Authors:** Ryan N McIlwain, Andrew J Heflin, Sharon Maina, William A Cutchen, Tyler C McDonald

**Affiliations:** 1 Department of Medicine, Frederick P. Whiddon College of Medicine, University of South Alabama, Mobile, USA; 2 Department of Orthopaedic Surgery, University of South Alabama, Mobile, USA

**Keywords:** caffey's disease, fetal bone disease, infantile cortical hyperostosis, neonatal bone disorders, periosteal reaction, prenatal cortical hyperostosis

## Abstract

Caffey’s disease, or infantile cortical hyperostosis, is a rare disorder characterized by subperiosteal new bone formation and diffuse soft tissue inflammation in infants younger than six months. A much rarer prenatal form presents before birth with diffuse, symmetric cortical thickening and often results in perinatal death, especially when it develops early in gestation. The severe form may be associated with pulmonary hypoplasia, hepatic dysfunction, and high neonatal mortality. We report the case of a premature infant born at 27 weeks’ gestation to a 36-year-old mother with chronic hypertension. Prenatal ultrasound demonstrated polyhydramnios, fetal hydrops, and abnormal long-bone ossification. Postnatal radiographs showed diffuse “cotton-wool” periosteal reactions involving all long bones and ribs, consistent with prenatal Caffey’s disease. Differential diagnoses such as osteogenesis imperfecta, congenital infection, and skeletal dysplasia were excluded through laboratory and genetic testing. The infant required prolonged ventilatory support but gradually improved and was discharged after a 19-week neonatal intensive care stay. At one-year follow-up, imaging showed near-complete remodeling of affected bones with only mild humeral deformity, and by two years, the child demonstrated normal growth, ambulation, and complete radiographic resolution. With diagnosis at 27 weeks, this case highlights one of the earliest documented survivors of severe prenatal Caffey's disease and suggests that once complications of prematurity are managed, the skeletal manifestations can resolve spontaneously. Awareness of this condition may help avoid misdiagnosis and guide appropriate counseling and follow-up.

## Introduction

Caffey’s disease, or infantile cortical hyperostosis (ICH), is a disorder characterized by spontaneous subperiosteal bone formation and diffuse inflammation [1]. An autosomal-dominant inheritance associated with a Type I collagen mutation (COL1A1) has been described [2]. It is hypothesized that alterations in Type I collagen impair extracellular matrix components and alter cell signaling, propagating a proliferative cell response [3]. It typically manifests in newborns before six months of age with global soft tissue swelling and is identified by the characteristic “patchy” areas of bone growth on radiographs, which are prone to spontaneous dissolution and recurrence. ICH is a self-limiting condition with typical resolution by three years [4,5].

There is a much rarer form, prenatal cortical hyperostosis (PCH), which is a distinct entity. Compared to ICH, this form is more diffuse, non-inflammatory, and the pathogenetic mechanism is less defined [2]. Osseous involvement is symmetric, manifesting as cortical bone thickening, or “cotton-wool” appearance, and angulated long bones are more common [6]. Most cases are lethal in the neonatal period (especially those with onset prior to 35 weeks) often due to underdeveloped lungs and/or liver failure [7-9]. An autosomal recessive mechanism has been theorized, though the COL1A1 mutation implicated in ICH appears to be absent in many cases of PCH [2]. A recent case report linked PCH to de novo mutations in IFITM5, a gene associated with severe osteogenesis imperfecta [10]. Despite this, the pathogenetic mechanism of disease remains largely unknown.

In this report, we present the case of a premature infant diagnosed with PCH who survived and showed a complete resolution of the characteristic osseous findings by two-year follow-up. This case is particularly notable, as the limited existing literature on PCH primarily describes infants who do not survive the neonatal period. As such, little is known about the natural progression of this rare disease.

## Case presentation

The patient was born at 27 weeks' gestation to a 36-year-old mother with chronic hypertension and late prenatal care. She had four prior uncomplicated pregnancies, resulting in healthy children. Prenatal ultrasound demonstrated polyhydramnios, micrognathia, frontal bossing, hydrops, and abnormal long bone ossification. At initial presentation, the newborn had a grossly distended abdomen and bilateral upper extremity deformities. Hypotonia and poor inspiratory effort led to intubation, complicated by retrognathia and a difficult airway. After improvement with administration of airway surfactant and abdominal paracentesis, the infant was weaned to high-frequency jet ventilation in the neonatal intensive care unit (NICU).

**Figure 1:**
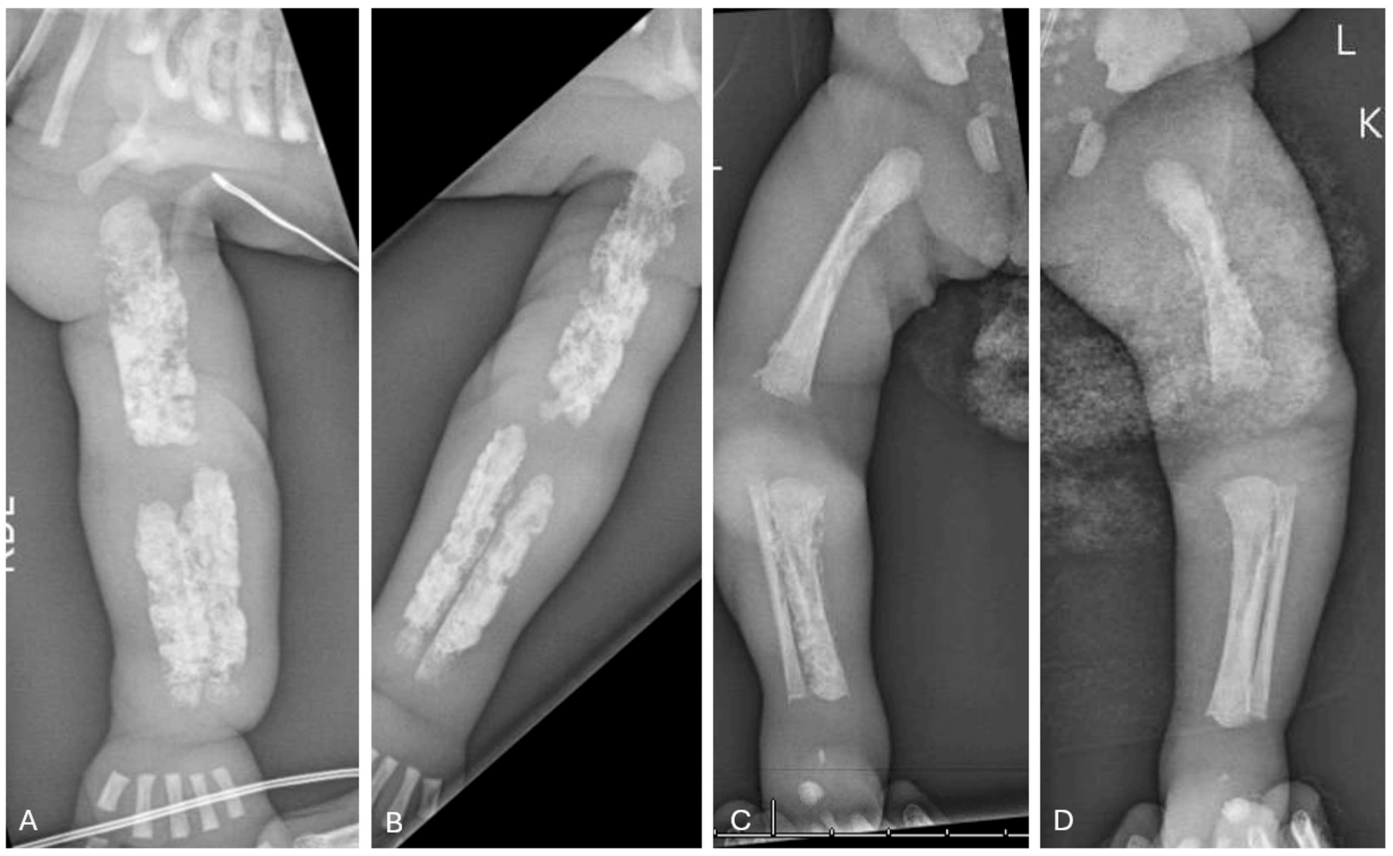
Radiographs of right (A) and left (B) humeri as well as right (C) and left (D) femora at birth. Radiographs demonstrate diffuse, permeative "cotton wool" appearance of the long bones as well as the partially-imaged ribs.

**Figure 2:**
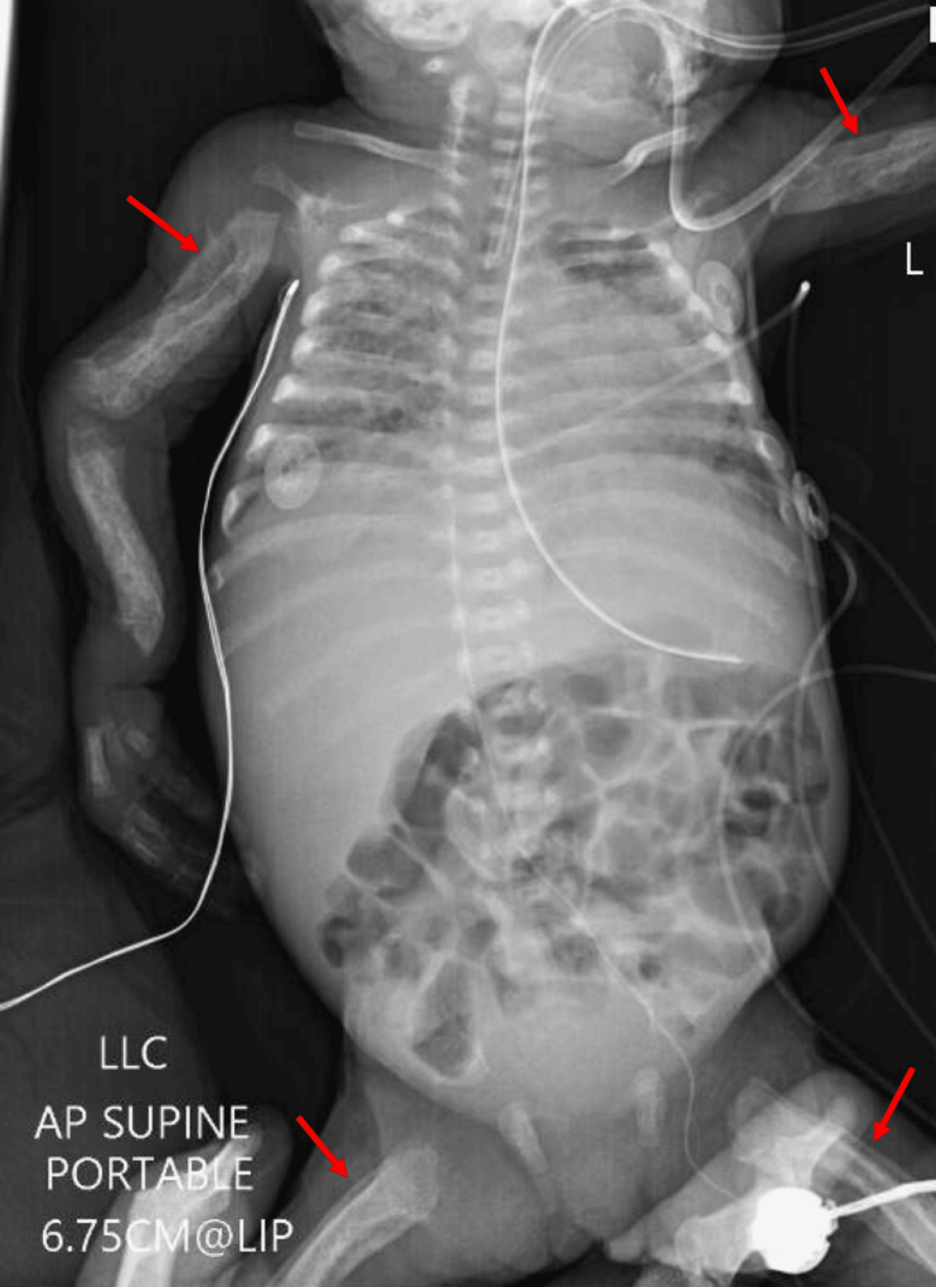
Babygram at age three weeks. Note the "double cortex" showing remodeling periosteum of the visualized portions of the long bones (red arrows show representative examples).

**Figure 3:**
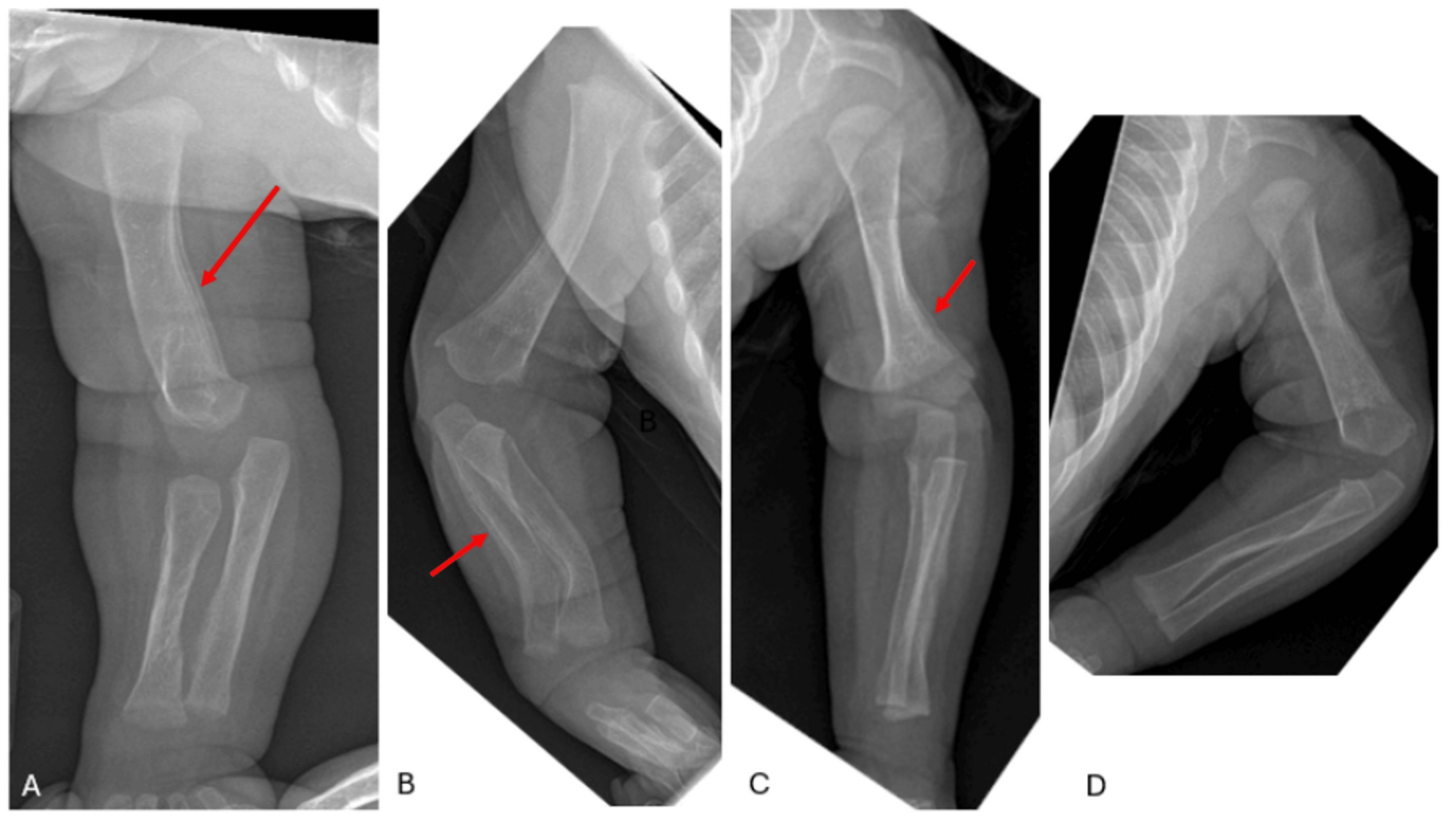
Right anteroposterior (A), right lateral (B), left anteroposterior (C), and left lateral (D) upper extremity radiographs at age five months. There is dramatic improvement in the appearance of the long bones with residual periosteal reaction (red arrows denote representative examples).

**Figure 4:**
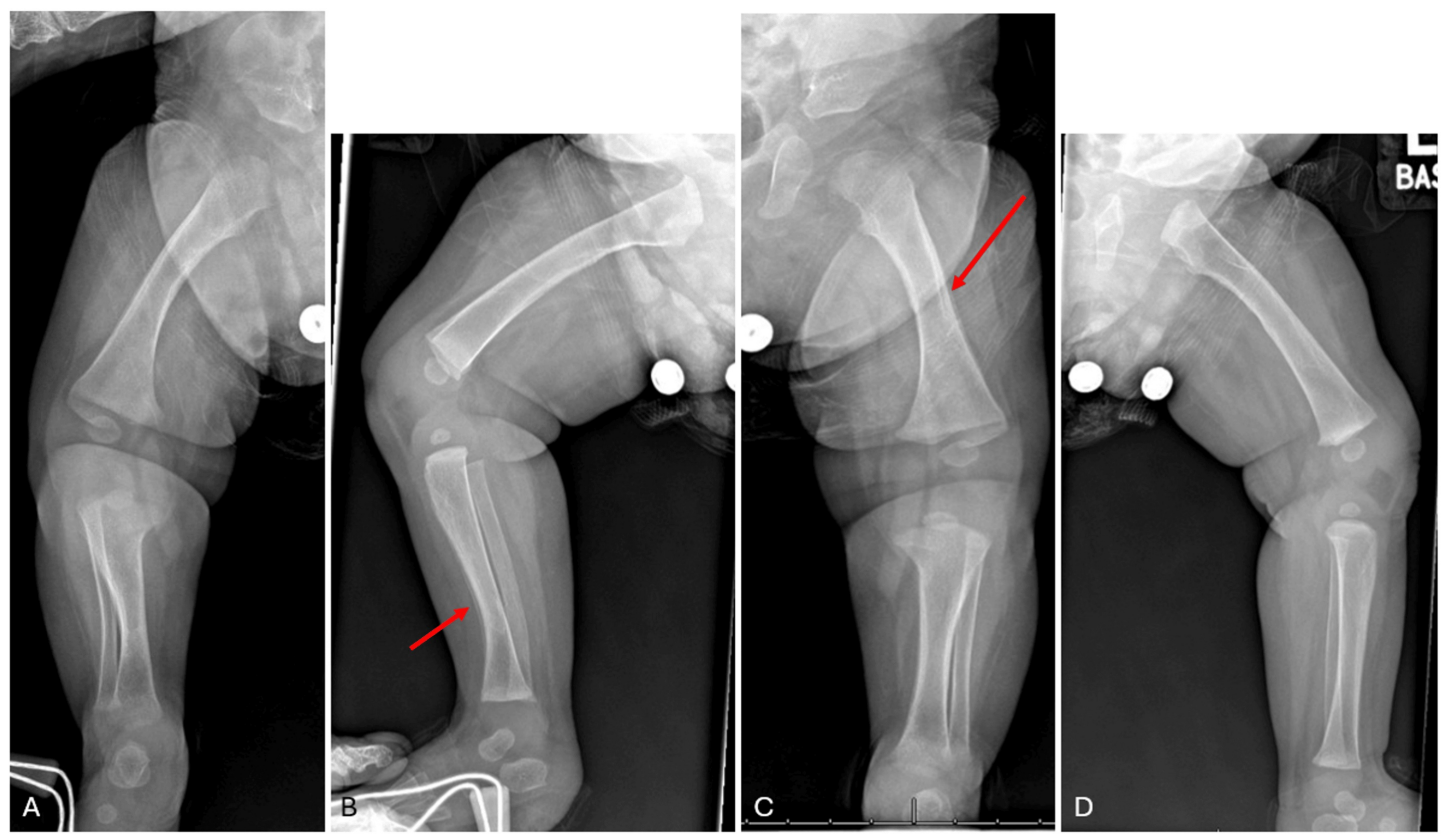
Right anteroposterior (A), right lateral (B), left anteroposterior (C), and left lateral (D) lower extremity radiographs at age five months. There is dramatic improvement in the appearance of the long bones with residual periosteal reaction (red arrows denote representative examples).

Initial full-body radiographs demonstrated diffuse “cotton-wool” periosteal reactions in all extremities and ribs (Figure 1A-D). Calcium, phosphate, and alkaline phosphatase (ALP) levels, as well as inflammatory markers, were normal. Osteomyelitis, metabolic diseases, and other self-limiting inflammatory processes were ruled out. Congenital syphilis, skeletal dysplasia, and osteogenesis imperfecta (OI) were also considered in the differential diagnosis but were excluded after a negative skeletal dysplasia panel (including COL1A1) and negative quantitative rapid plasma reagin (RPR). Lethal hypophosphatasia was considered less likely in the absence of skull and vertebral body involvement. With the history of polyhydramnios, fetal hydrops, and radiographic appearance, PCH was diagnosed. By three weeks, the patient’s bones had begun remodeling and maturing compared to initial radiographs (Figure 2). The patient had a tenuous 19-week NICU stay complicated by respiratory distress and feeding intolerance but ultimately improved and was discharged home. At the first outpatient follow-up visit in the pediatric orthopaedic clinic (at age five months), x-rays revealed drastic improvement with residual periosteal reaction (Figure 3A-D and Figure 4A-D).

**Figure 5:**
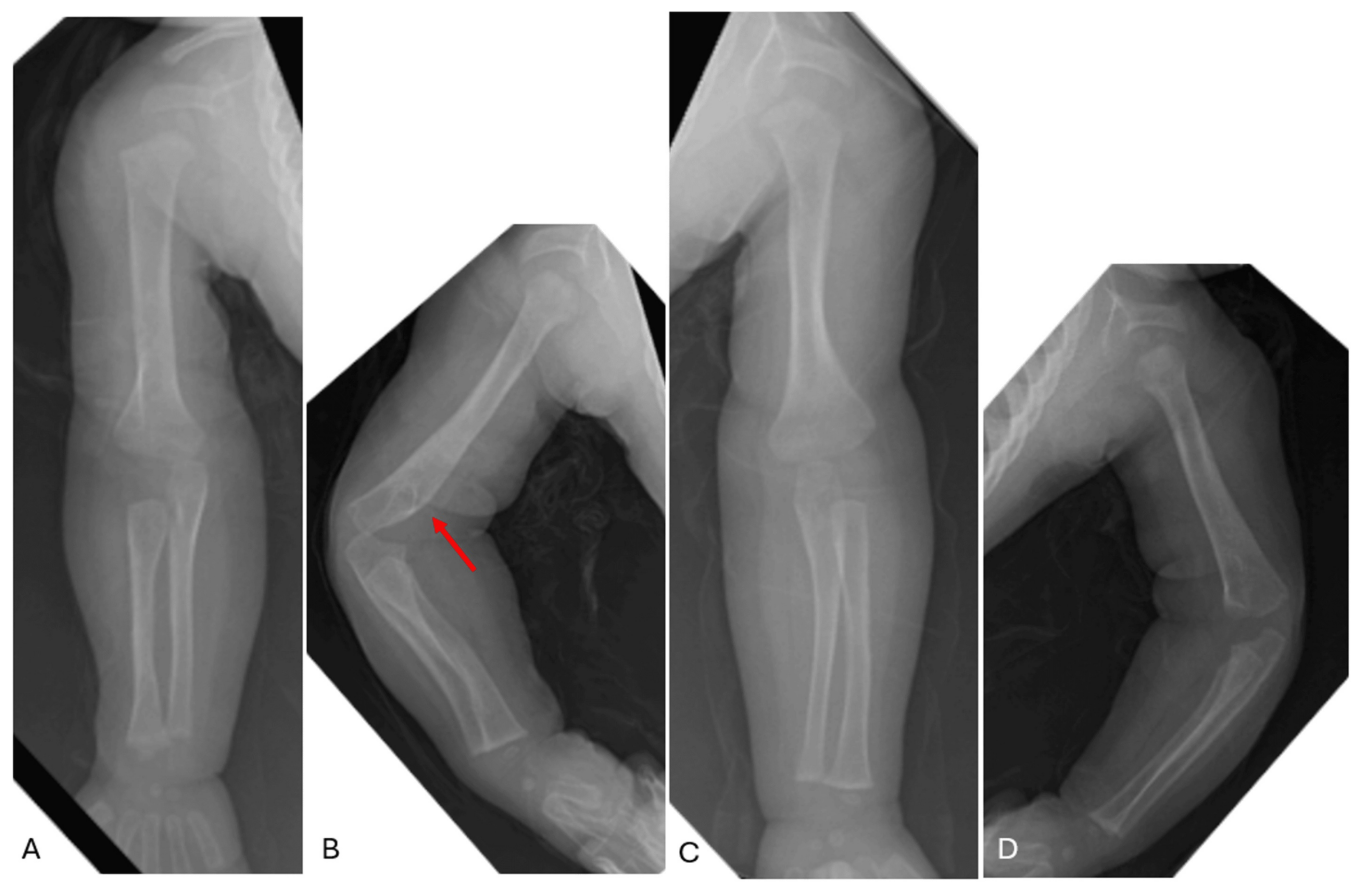
Right anteroposterior (A), right lateral (B), left anteroposterior (C), and left lateral (D) upper extremity radiographs one year of age. The long bones appear normal with no periosteal reaction. Red arrow denotes the mild procurvatum deformity of the right distal humerus.

**Figure 6:**
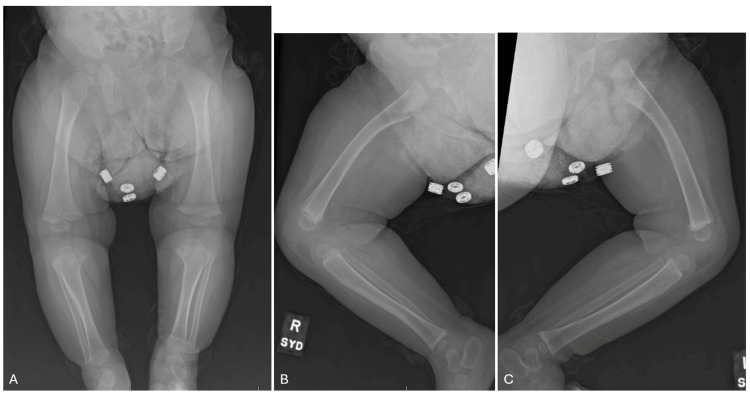
Anteroposterior (A), right lateral (B), and left lateral (C) radiographs at one year of age. The long bones appear normal with no periosteal reaction.

**Figure 7:**
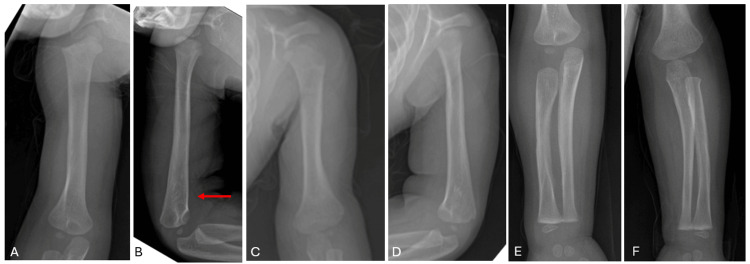
Right anteroposterior (A), right lateral (B), left anteroposterior (C), and left lateral (D) humerei as well as right (E) and left (F) anteroposterior forearm radiographs at two years of age. The long bones appear normal, and the procurvatum deformity of the right distal humerus seen in Figure 5B (in the area denoted by the red arrow) has resolved.

**Figure 8:**
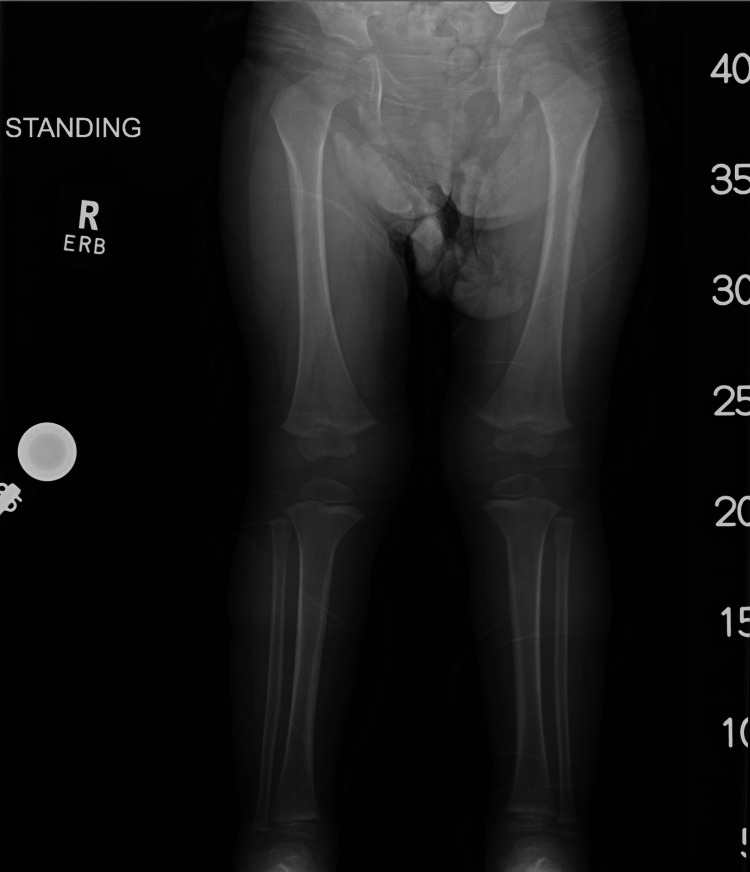
Standing anteroposterior hip-to-ankle radiographs of bilateral lower extremities at two years of age showing normal appearance of the long bones.

Outpatient follow-up at one year demonstrated resolution of the osseous findings on imaging, with normal-appearing bones except for a mild procurvatum deformity of the right distal humerus (Figures 5A-D and Figure 6A-C). The patient was able to stand on her own with no support. At two-year follow-up, she was asymptomatic, ambulating normally for age, and meeting developmental milestones. Upper and lower extremity radiographs demonstrated a complete resolution of prior periosteal reactions (Figures 7A-F and Figure 8). The procurvatum deformity of the right humerus had resolved. Table 1 shows representative chronologic radiographic and clinical findings throughout the patient's first two years.

**Table 1:** Chronological milestones demonstrating resolution of disease

Age	Birth	3-weeks	5-months	1 year	2 years
Radiologic findings	Diffuse periosteal reaction in extremity long bones	“Double cortex” sign indicating remodeling periosteum (Figure 2)	Complete resolution of “cotton-wool” appearance with residual periosteal reactions (Figure 3A-D and Figure 4A-D)	Normal appearing long bones	Normal appearing long bones
	Bilateral humeri angulations (Figure 1A-D)			Mild procurvatum deformity of right distal humerus (Figure 5A-D and Figure 6)	Resolution of procurvatum deformity (Figure 7B)
Clinical status	Respiratory insufficiency, ascites, hypotonia, feeding intolerance	Weaned off ventilator	Meeting developmental milestones	Meeting developmental milestones	Meeting developmental milestones, asymptomatic
		Discharged home	Improved feeding intolerance	Able to stand without support	Ambulating appropriately for age

## Discussion

This case report describes a newborn diagnosed with the prenatal form of Caffey’s disease, with a complete resolution of osseous findings by two-year follow-up. Caffey’s disease can be divided into two forms. The infantile form (ICH) is more common and typically manifests between nine and 12 weeks postnatally [4]. The prenatal form (PCH) is exceedingly rare, with around 50 cases described in the literature [11]. Other than age at onset, radiographic findings help delineate the two entities. ICH is a widely inflammatory process with characteristic cortical bone thickening preceded by local soft-tissue swelling [4]. Recent theories suggest a mutation in the collagen structure accomplishes two concomitant events, which explain this clinical presentation: (1) upregulation of the COX2/PGE inflammatory cascade and (2) disruption of extracellular membrane interactions with subsequent altered cell signaling and proliferation [3]. Classically, a fetus with PCH will demonstrate a symmetric, “moth-eaten” or “cotton wool” appearance in long bones with potential angulations. The mandible, ribs, and scapula may be involved as well [6]. PCH is often misdiagnosed as osteogenesis imperfecta, as the angulated appearance can be mistaken for a fracture. Given the inflammatory etiology, other common misdiagnoses include osteomyelitis and congenital syphilis due to the diffuse cortical reaction and long bone involvement [7,11]. This case aligns with prior findings, as diffuse “cotton-wool” reactions and angulated upper extremity long bones were noted on prenatal radiographs.

PCH can be further divided into mild and severe forms based on onset before or after 35 weeks. In a review of published cases, Schweiger et al. reported 26 cases of PCH categorized as “severe.” Most of these patients died in utero and 11 out of the 26 were stillborn with diffuse involvement of the long bones. Of the 15 liveborn, six died in the early neonatal period from respiratory insufficiency [7]. Building on this, Nemec et al. reviewed a cohort of 20 patients with mild or severe form PCH where 10 succumbed in the prenatal period [6]. Given the high mortality rate observed by Schweiger and Nemec et al. was secondary to severe prematurity, the progression and mortality risk of the disease itself are not well known [8,9,12]. There are select reports describing a spontaneous resolution of the severe form; Hoen et al. described two cases where bony angulations were apparent at 26 and 28 weeks [13]. Diagnosis was made postnatally in these two cases, whereas PCH was diagnosed at birth in this case. The proposed explanation for the high mortality involves a sequence of events initiated by a disruption in bone marrow hematopoiesis. This alteration in osmotic pressure promotes diffuse anasarca and polyhydramnios, with subsequent hepatomegaly and limited fetal movement contributing to pulmonary hypoplasia [14,15]. In this report, the patient’s presentation is classified as severe PCH with clinical manifestations at birth (27 weeks). Additionally, this patient presented with fetal hydrops and poor respiratory effort similar to other described cases of severe PCH.

With the diagnosis of PCH at 27 weeks, this case represents one of the earliest documented survivors of severe PCH who underwent complete radiographic resolution. These findings support the notion that PCH may be self-resolving in select survivors once prematurity-related complications are managed successfully. This case may also help redefine counseling parameters, as the chance for survival could influence pregnancy decision-making after prenatal diagnosis.

## Conclusions

This case represents one of the few documented survivors of severe prenatal-onset Caffey’s disease and highlights that even the most critical presentations may follow a benign, self-resolving course when neonatal complications are overcome. By demonstrating complete radiographic and functional recovery by two years of age, this report expands the limited understanding of disease progression and prognosis in severe PCH. Further, this case highlights the importance of distinguishing PCH from other highly lethal skeletal dysplasias to optimize neonatal outcomes. Early recognition is essential to avoid misdiagnosis, guide family counseling, and prevent unnecessary interventions. Continued reporting of similar cases is needed to clarify the underlying mechanisms, refine prognostic expectations, and better distinguish lethal from survivable phenotypes.
